# Interaction of ATP with fibroblast growth factor 2: biochemical characterization and consequence for growth factor stability

**DOI:** 10.1186/1471-2091-12-14

**Published:** 2011-03-29

**Authors:** Karsten Rose

**Affiliations:** 1 Institut für Pharmazeutische und Medizinische Chemie, Westfälische Wilhelms-Universität Münster, Hittorfstr. 58-62, 48149 Münster, Germany

## Abstract

**Background:**

Fibroblast growth factor 2, a well-characterized heparin-binding growth factor, is involved in many biological processes like embryogenesis, cell proliferation and angiogenesis. However, this growth factor is very unstable and shows rapid degradation in aqueous solution. Beside the well-known stabilization of FGF2 by heparin or heparan sulphate, the recently discovered binding to ATP also shows a stabilizing and protective effect on this growth factor.

**Results:**

Here we determined the dissociation constant of ATP on FGF2 by equilibrium microdialysis (K_D_: 59.8 μM) and analyzed the impact of this binding on secondary structure by CD-spectroscopy. ATP-binding to FGF2 significantly changed the secondary structure of this growth factor with a shift to random coil structure elements. We also analyzed the influence of this binding on the stability of FGF2 in aqueous solution over a period of 2 h. While the amount of untreated FGF2 is reduced drastically over this period of time, ATP-binding reduces the degradation considerably.

**Conclusions:**

Taken together, our data suggest an important role of ATP in FGF2-stabilization beside the well known-role of heparin and heparan sulphate.

## Background

Some groups of protein families are known to bind ATP, for example kinases and ABC transporters. Both protein families possess a conserved ATP-binding domain called P-loop (formerly Walker motif A: GXXG(X)XGKT(X); [1,2]. However, ATP-binding which is not based on a specific ATP-binding domain, is described for many other proteins like lactoferrin [3], bovine serum albumin [4] or gelsolin [5]. Recently, the interaction of ATP with growth factors was discovered [6]. The physiological role of this ATP-binding to growth factors remained unclear. However, binding of ATP to NGF and FGF2 seems to be essential for their neuroprotectivity as demonstrated with hippocampal neuronal cells [7,8]. The heparin binding domain (HBD) of both growth factors is important for ATP-binding [7,9] although crystallographic data of the growth factor/ATP-complexes are not yet available.

Complex formation of ATP with NGF and FGF2 is probably based on ionic interaction of the negatively charged phosphate residues of ATP with positively charged arginine and lysine residues located in the HBD of NGF and FGF2, respectively. This is similar to the interaction of sulphate residues of heparin or heparan sulphate-proteoglycans (HSPGs) with heparin-binding growth factors like fibroblast growth factors, VEGF, NGF or PDGF [10,11]. Additionally, binding of ATP to FGF2 stabilizes the growth factor and protects it from thermal degradation and proteolytic cleavage with trypsin, neutrophil elastase (NE) or plasmin [12].

In this paper we determined the dissociation constant (K_D_) of ATP-binding to FGF2 and analyzed the impact on the conformation of the protein. Furthermore, we investigated the consequence of this nucleotide binding on FGF2 stability in aqueous solution to get a more detailed insight into this probably important protein-ligand interaction.

## Methods

### Equilibrium-microdialysis

Rapid equilibrium dialysis inserts (RED device inserts, Pierce, IL, USA) were used along with Teflon Base Plate (Pierce, Il, USA) for equilibrium dialysis experiments. These inserts contain two 750-μL chambers separated by 8,000 Da molecular mass cut-off. One chamber was filled with FGF2 (10 μM) in 25 mM Tris-HCl, pH 7.5 (550 μL), and the other with various concentrations of ATP in 25 mM Tris-HCl, pH 7.5. The dialysis inserts were rotated at room temperature with a speed of 250 rpm and equilibrium was reached within 4 h as monitored by counting an aliquot from both sides of the membrane at different time points during dialysis. After 4 h, 50 μL aliquots of the dialysis chambers were removed and kept on ice. ATP was determined immediately. The experiments were performed in triplicate.

### CD measurement

Far-UV spectra (195-250 nm) were recorded at FGF2 concentration of 25 μM with a Jasco-J600 spectropolarimeter at room temperature in a 0.1-cm cell with 4 accumulations. Measurements were performed in 25 mM Tris-HCl (pH 7.5), and all spectra were corrected for buffer and nucleotide background. The results are expressed as ellipticities related to the mean residue weight of amino acids as described before [7].

### Chemical cross-linking experiments

For cross-linking studies, FGF2 (5 μM) in Tris-HCl (pH 7.5) was incubated with or without nucleotides (1 mM), heparin (10 μg/mL), and/or protamine (20 μg/mL) at 37°C for 15 min. 1 mM bis(sulphosuccinimidyl)suberate (BS^3^) was added and the reactions were incubated for additional 1 h at 20°C. Reactions were stopped by adding SDS-loading dye and subsequent heating at 95°C for 5 min. Proteins were separated by SDS-PAGE and visualized by silver staining.

### Degradation of FGF2 and FGF2(K134A) by NE

Degradation of FGF2 and FGF2(K134A) by NE was performed as described previously [12]. FGF2 (with his-tag) and FGF2(K134A) (with his-tag) were expressed and purified as described previously [13].

### Elisa

FGF2-ELISA was performed according to the supplier's instructions (Ray Biotech, GA, USA). In brief, 100 μL of each sample containing FGF2 were added to a 96-well plate, coated with antibody specific for human FGF2, and incubated at 4°C over night. Wells were washed 4 times and then incubated with biotinylated anti-human FGF2 antibody for 1 h at RT. After additional washing (4 times) streptavidin solution was added to each well and incubated for 45 min. After washing (4 times) 3,3',5,5'-tetramethylbenzidine-solution was added to the wells and incubated for 30 min, the reactions were stopped and the absorbance was read at 450 nm immediately.

### Measurement of ATP content

ATP concentration was determined luminometrically as described previously [7].

## Results

### Dissociation constant of ATP-binding to FGF2

**Figure 1:**
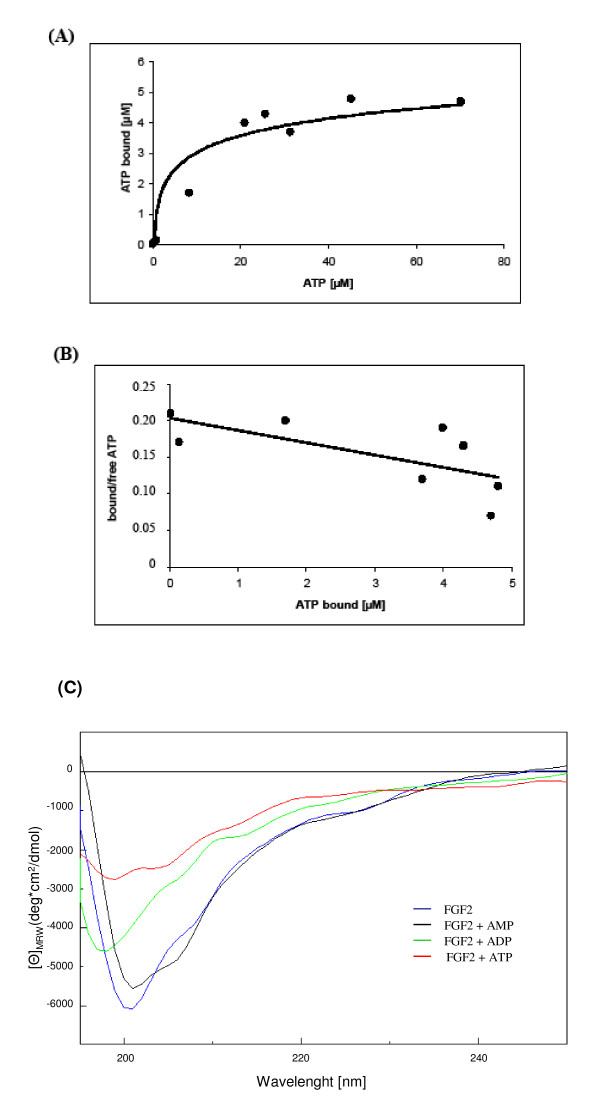
**Binding of ATP to FGF2 and nucleotide-induced conformational change**. (A) The binding of ATP to FGF2 was determined by equilibrium microdialysis. FGF2 (10 μM) was incubated with different concentrations of ATP in rapid equilibrium dialysis inserts in a Teflon Base Plate for 16 h at 20°C. ATP in the dialysis chambers was determined using ATP Kit SL and a LKB Wallac luminometer. (B) The data were analyzed in a Scatchard plot using a calculated amount of bound and free ATP to determine K_D _of FGF2/ATP-complex. The equation was used to calculate the number of binding sites of 1.2 (y-value is set = 0) and the K_D _value of 59.8 μM (the reciprocal of the negative gradient). (C) CD spectra of FGF2 (25 μM) measured in Tris-HCl (pH 7.5). Effects of different nucleotides (100 μM, respectively) are shown. The ordinate axis presents the molecular ellipticity.

Previous work has shown that ATP can bind to FGF2 [9,14]. To characterize this binding of ATP to FGF2 in more detail, we used equilibrium microdialysis to directly examine the ATP-binding properties of the growth factor. Equilibrium microdialysis was performed with 10 μM FGF2 in the reaction chamber. As ATP concentrations increased from 1 to 80 μM, the amount of ATP bound to FGF2 increased as well reaching a maximum level and indicating a saturation binding behavior (Figure 1A). Consistent with the radiolabeling experiments [9] and MALDI-TOF data [14,15], FGF2 (10 μM) clearly bound ATP at concentrations between 1 and 80 μM. ATP binding data were replotted in a Scatchard plot and the binding constant was calculated. The linear Scatchard plot gave a value of 1.2 for the number of independent binding sites, indicating that one molecule ATP binds to one molecule FGF2 (Figure 1B). The calculated dissociation constant (K_D_) is 59.8 μM.

### Conformational change of secondary structure of FGF2 after ATP-binding

We collected CD spectra of untreated FGF2 only and FGF2 preincubated with nucleotides to investigate putative conformational changes of the secondary structure of the FGF2/ligand-complex. The CD-spectrum of FGF2 (concentration: 25 μM) showed a curve progression characteristic for a protein rich in β-structure (Figure 1C). The signal decreased from 230 nm onward reaching a broad minimum around 205 nm to increase again to the base level, as it has been reported previously for NGF [7]. In contrast to untreated FGF2, FGF2 incubated with ATP (100 μM) showed a clear decrease of the amplitude of the broad minimum (Figure 1C) and no increase afterwards to the base level at 195 nm. This indicates a decrease of percentage of β-structure and finally a conformational change of FGF2 after interaction with this nucleotide. In contrast, the CD-spectrum of FGF2 did not significantly change after addition of AMP (100 μM). After addition of ADP (100 μM) the CD-spectrum changed only moderately compared to untreated FGF2.

### Influence of ATP on dimerization behaviour of FGF2

**Figure 2:**
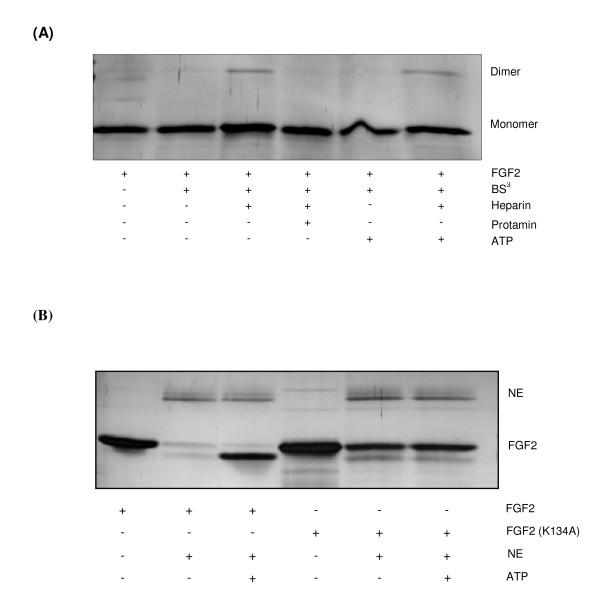
**FGF2 monomers and dimers after cross-linking and variant forms of FGF2 after proteolytic degradation**. (A) FGF2 (1.5 μg) in Tris-HCl (pH 7.5) was incubated with 1 mM ATP and 10 μg/mL heparin and 20 μg/mL protamine at 37°C. After 15 min, 1 mM BS^3^-crosslinker was added and the samples were incubated for 1 h at 20°C. Reactions were stopped by addition of SDS-loading dye and boiling. The proteins were separated by 15% SDS-PAGE and visualized by silver staining. (B) Two μg FGF2 (K134A) in Tris-HCl (pH 7.5), were incubated with ATP (100 μM) or without ATP at 37°C. After 15 min FGF2 and FGF2 (K134A) were incubated with 200 ng NE at 37°C for 2 h. The reactions were stopped by addition of SDS-loading dye and boiling. The samples were separated by SDS-PAGE and protein was visualized by silver staining.

Previous studies reported dimerization of FGF2 and additional receptor binding induced by heparin [16]. Thus, we were highly interested in finding out whether ATP binding or subsequent structural changes could also promote FGF2 dimerization. Addition of 1 mM ATP and BS^3 ^(Figure 2A, lane 5) did not induce dimerization whereas addition of heparin and BS^3 ^did (Figure 2A, lane 3). However, co-incubation of FGF2 with heparin (10 μg/mL), ATP (1 mM) and BS^3 ^(Figure 2A, lane 6) did promote formation of dimers with a similar dimer/monomer ratio as observed for incubation with heparin and subsequently BS^3^-crosslinking only (Figure 2A, lane 3).

### Stability of FGF2 and FGF2 (K134A) against proteolytic digestion

Recently, we reported that a variant form of FGF2 (FGF2-Quadro, [12]) with mutations in the heparin binding domain did not show enhanced stability against trypsin degradation after incubation with ATP because of its reduced ATP-binding capacity. Here, we investigated the stability of mutant FGF2(K134A) against degradation by neutrophil elastase (NE). The K134A variant showed a partial stability against degradation by NE independent of this protein being preincubated with ATP or not (Figure 2B). Addition of ATP did not additionally protect FGF2(K134A) from protease digestion as revealed by silver staining. Interestingly, the ATP-binding capacity of FGF2(K134A) was only moderately reduced compared to the wild-type [9].

### Stability of FGF2 in aqueous solution is increased after ATP-binding

**Figure 3:**
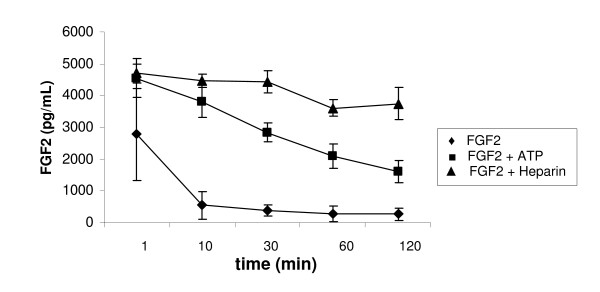
**Stability of FGF2 in aqueous solution**. FGF2 (5 ng/mL) was incubated in 25 mM Tris, HCl (pH 7.5) at 37°C with or without preincubation with ATP (200 μM) or heparin (10 μg/mL), respectively. Samples were collected at different points of time and FGF2-concentration was determined with Human bFGF ELISA Kit (RayBiotech, USA) in triplicate.

ATP-binding to FGF2 increased the stability of this growth factor against proteolytic degradation as described previously [12]. Here, we wanted to investigate whether ATP protects the growth factor also from physical inactivation, meaning the stability in aqueous solution. Therefore, we performed stability experiments of FGF2 in Tris-buffered aqueous solution at 37°C and subsequently determined FGF2-concentration by ELISA. We incubated untreated FGF2, preincubated with ATP (200 μM) or preincubated with heparin (10 μg/mL) at 37°C for 2 h. Figure 3 shows that the concentration of unprotected FGF2 decreased drastically already after 10 minutes of incubation from 5 ng/mL to about 0.5 ng/mL, whereas the concentration of FGF2, preincubated with heparin, only marginally decreased over the period of 2 h from about 5 ng/mL to about 4 ng/mL. The stability of FGF2, preincubated with ATP, decreased from about 5 ng/mL to about 3 ng/mL after 30 min and to about 2 ng/mL after 120 min.

Additionally, we measured the ATP concentration luminometrically at different points of time in the experiment with FGF2 preincubated with ATP. The ATP-concentration did not change significantly indicating that ATP was stable over the observed period of two hours (data not shown).

### Stability of FGF2 depends on number of phosphate residues of used stabilizers

**Figure 4:**
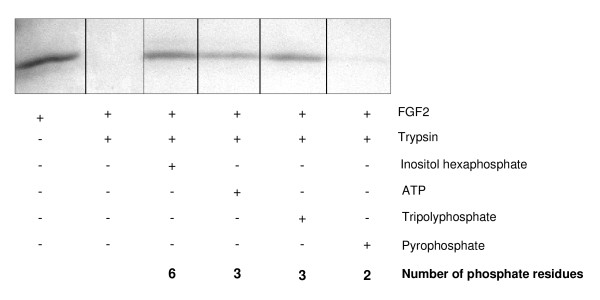
**Stability of FGF2 depends on the number of phosphate residues of bound stabilizers**. FGF2 (5 μM) was preincubated with different stabilizers (100 μM each) at 37°C for 15 min. The complexes were then treated with trypsin (50 ng) at 37°C for 3 h. The samples were separated by SDS-PAGE and protein was visualized by Coomassie-staining.

In order to investigate the importance of the number and the position of phosphate residues of putative FGF2-stabilizers, we protected FGF2 from trypsin degradation using substances with a different number of phosphate residues in the range of 2 (pyrophosphate) to 6 (inositol hexaphosphate). FGF2 was preincubated with each substance to form FGF2/ligand complexes. Then, trypsin was added and the samples were incubated for 3 h. It was generally observed that with an increasing number of phosphate residues the protective effect on FGF2 against tryptic degradation increased respectively (Figure 4). Inositol hexaphosphate (6 phosphate residues) revealed a strong protection of the growth factor from tryptic digestion, whereas ATP and tripolyphosphate (3 phosphate residues) showed a slightly decreased protective effect. Pyrophosphate (2 phosphate residues) only had a marginal protective effect on FGF2 degradation under these experimental conditions.

## Discussion and conclusions

Besides the large groups of ATP-binding proteins, for example the kinases and the ATPases with more or less defined ATP-binding domains (P-loop, Kinase 1 or Kinase 2), some other ATP-binding proteins are described in the literature, e.g. ABC transporters, metallothionein, chaperons and actins which lack such a domain [17]. Growth factors have been identified as ATP-binding proteins only recently [6]. Nerve growth factor (NGF), brain-derived growth factor (BDNF), vascular endothelial growth factor (VEGF; personal communication R. Gast) and FGF2 are associated with ATP which is very likely to be important for their biological activity.

In this work, the binding affinity of ATP to FGF2 was determined by equilibrium microdialysis. The K_D _value of 59.8 μM showed only moderate binding of ATP to FGF2 which is contradictory to the binding affinities described for most kinases/ATP- and ATPases/ATP-complexes. This moderate binding constant is well observed to be not consistent with the ATP-concentration in the low nmolar range necessary for neuroprotective activity of FGF2 in cell culture experiments demonstrated recently [8]. Based on the experiments described in this paper, a high affine binding domain of ATP besides the 59.8 μmolar binding domain is very likely to be present. However, the K_D _values of other ATP-binding proteins, e.g. gelsolin (K_D _32 μM, [18]), p-glycoprotein (K_D _200 μM; [19]) and metallothionein (K_D _176 μM; [20]), were in a similar range like FGF2. The value of 1.2 for the number of independent ATP-binding sites of FGF2 showed that one molecule of FGF2 binds one molecule of ATP. This is in line with MALDI-TOF experiments where binding of one molecule of ATP to one molecule of FGF2 was demonstrated [14,15]. Only in the presence of magnesium two molecules of ATP were able to bind to FGF2. Comparing the K_D _value of FGF2/ATP (K_D_: 60 μM) with FGF2/HSPGs (K_D_: 1-50 nM, [21,22]) demonstrated that the binding of HSPGs to FGF2 was preferred.

Although the dissociation constant of ATP-binding to FGF2 was rather high, this binding leads to a significant change in the secondary structure of FGF2 as demonstrated by CD-spectroscopy (Figure 1C). Thereby the amplitude of structural change depends on the number of phosphate residues of the nucleotides (ATP > ADP > AMP). Interestingly, the conformational change after ATP-binding to FGF2 was more distinct than that of heparin [23,24] and may explain the physiological importance for neuroprotective activity of FGF2 [8].

It has been known for many years that binding of heparin or HSPGs to FGF2 forces the dimerization of the growth factor and that this is important for interaction with its specific receptors ([16]; Figure 2A). In contrast to heparin, ATP did not lead to a dimerization of FGF2 as observed in cross-linking experiments (Figure 2A, lane 5). It could be speculated that ATP is not involved in receptor activation by dimerization. More important seems to be the protective effect of this abundantly present nucleotide on FGF2. This is in line with recent data showing that the main role of heparin or HSPGs in FGF1-induced signaling is to protect this naturally unstable protein from heat and proteolytic degradation [25].

The ATP binding capacity of the mutant FGF2 (K134A) is reduced by about 20% compared to wt-FGF2 [9]. Adding ATP to FGF2 (K134A) did not show additional protective effects against degradation by NE (Figure 2B). Furthermore, mutant FGF2 (K134A) did not show neuroprotective activity [13]. It could be speculated that the not observed protectivity of ATP on FGF2 (K134A) is important for the loss of neuroprotectivity of this variant form of the growth factor [8].

Besides the protection of FGF2 by bound ATP from proteolytic digestion [12], we demonstrated in this work that ATP additionally protects FGF2 from physical degradation. It is known that FGF2 is rapidly degraded when incubated in aqueous solution without stabilizers [26]. This is the main problem in the use of this important growth factor for medical applications. Observing the stability of FGF2 in buffered aqueous solution over a period of 2 hours clearly showed a protective effect of ATP bound to FGF2 (Figure 3). However, this protective effect was not as strong as observed with heparin; heparin protected FGF2 nearly completely from physical degradation (Figure 3). This decreased effect on the stability of FGF2 by ATP compared to heparin might be explained by the higher dissociation constant of ATP (59.8 μM) in contrast to heparin (1-50 nM).

The CD-data clearly demonstrate that the number of phosphate residues of the nucleotide (AMP, ADP, and ATP) is very important for the conformational change of the growth factor. Therefore we investigated the effect of FGF2-stabilization by different ligands exhibiting phosphate residues in the range of 2-6 (Figure 4). The results show that the number of negatively charged groups is important for the protective effect against proteolysis. However, thereby it was not taken into account that each substance has a different structure with different distances between the phosphate residues.

**Figure 5:**
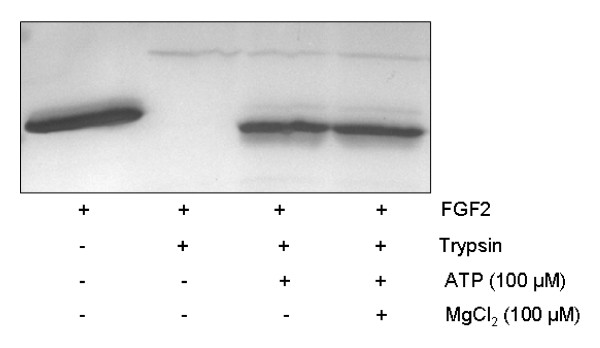
**Stabilization of FGF2 by ATP/Mg^2+^-complex**. FGF2 (5 μM) in Tris-HCl (pH 7.5) was incubated with or without ATP (100 μM), MgCl_2 _(100 μM) and trypsin (50 ng) at 37°C for 3 h. The samples were separated by SDS-PAGE and protein was visualized by silver-staining.

Taken together our results indicate that the binding affinity of ATP to FGF2 is moderate, but has a high impact on the secondary structure of the protein. This change in secondary structure might be the reason for the observed effect of ATP on the FGF2-stability, which could be classified between non-protected-FGF2 and a full-protection by FGF2/heparin-complex. Interestingly, the protective effect of ATP (0.1 mM) on FGF2 could also be detected after addition of 0.1 mM MgCl_2 _leading to the formation of the physiologically important ATP/Mg^2+^-complex (Figure 5). Such a ATP/Mg^2+^-complex (molar ratio 1:1) is the predominant form of extracellular ATP in tissue.

Taken together, a putative physiological role of FGF2-protection by ATP in cell culture and tissue is well conceivable and has to be proved in future studies.

## Abbreviations

FGF2: fibroblast growth factor 2; NGF: nerve growth factor; BDNF: brain-derived growth factor; VEGF: vascular endothelial growth factor; NE: neutrophil elastase

## Authors' contributions

KR carried out all experiments and drafted the manuscript.
